# Stress Assessment in *Caretta caretta* During the Rehabilitation Period

**DOI:** 10.3390/ani16101554

**Published:** 2026-05-20

**Authors:** Chiara Lomonaco, Giorgia Schiró, Paola Galluzzo, Rosaria Disclafani, Irene Vazzana, Salvatore Dara, Giuseppe Piccione, Vincenzo Monteverde, Claudia Giannetto

**Affiliations:** 1 Department of Veterinary Science, University of Messina, 98168 Messina, Italy; chiara.lomonaco1@studenti.unime.it (C.L.); giorgia.schiro@izssicilia.it (G.S.); giuseppe.piccione@unime.it (G.P.); claudia.giannetto1@unime.it (C.G.); 2 National Reference Center on Welfare, Monitoring and Diagnostics of Sea Turtle Diseases, 90129 Palermo, Italy; irene.vazzana@izssicilia.it (I.V.); salvatore.dara@izssicilia.it (S.D.); vincenzo.monteverde@izssicilia.it (V.M.); 3 Center for Sustainability and Ecological Transition, University of Palermo, 90133 Palermo, Italy

**Keywords:** *Caretta caretta*, sea turtle, stress, corticosterone, rehabilitation

## Abstract

The rehabilitation of the loggerhead sea turtle, *Caretta caretta*, involves stressors like handling and confinement. To assess physiological stress responses during rehabilitation, twenty-five *C. caretta* hospitalized at C.Re.Ta.M. were monitored over a two-month period at three time points (T0, T1, and T2). The cohort included 12 juveniles (CCL: 30.6 ± 5.7 cm) and 13 subadults (CCL: 52.5 ± 10.4 cm). Heterophil/lymphocyte ratios (H/L), corticosterone (CORT), glucose (Glu), creatine kinase (CK), and uric acid (UA) plasma concentrations were assessed. Two-way repeated-measure ANOVA revealed significant time effects on H/L ratio (*p* < 0.0001), CORT (*p* < 0.0001), Glu (*p* = 0.0002), CK (*p* < 0.0001), and UA (*p* < 0.05), with a significative group x time interaction observed for CK (*p* = 0.016), CORT (*p* = 0.006) and UA (*p* = 0.035). No group effect was observed in any of the data. In the juvenile group, H/L (*p* < 0.01) and CORT (*p* < 0.001) were significantly lower at T2 compared to T0. At the T0 point, CORT levels were significantly higher in juveniles compared to the subadult group. In subadults, significant decreases in H/L ratio (*p* < 0.001), Glu (*p* < 0.01), CK (*p* < 0.001), and UA (*p* < 0.05) were observed at both T1 and T2 relative to T0. At T0, CK levels were significantly higher in subadults compared to juveniles. No significant correlations were found between CORT and the other measured parameters. Our results suggest that the rehabilitation period is a safety period during which the animals reestablish their homeostasis despite captivity conditions. However, further studies are needed to define other causes of variations in stress levels in rehabilitating *C. caretta*.

## Simple Summary

Rehabilitation of the loggerhead sea turtle, *Caretta caretta,* involves several potential stressors, including handling, artificial feedings, and confinement within tanks. To evaluate how these animals cope with the recovery process, the stress levels of twenty-five *C. caretta* (12 juveniles and 13 subadults) housed at the C.Re.Ta.M. were monitored over a two-month period. Blood parameters analyzed were: heterophil/lymphocyte ratio, corticosterone, glucose, creatine kinase and uric acid. Our findings showed a significant decrease in almost all stress markers over time, particularly in subadults. Despite the constraints of captivity, these subjects were able to re-establish their homeostasis during their stay. These results suggest that the rehabilitation period in the rescue center effectively improves the health status of these animals. However, further studies are necessary to elucidate additional factors influencing stress levels in *C. caretta* during rehabilitation.

## 1. Introduction

The loggerhead sea turtle, *Caretta caretta* (Linnaeus, 1758), is a long-lived marine species widely distributed across the temperate and tropical zones of all the world’s oceans, as well as throughout the Mediterranean Sea [1,2]. Due to significant threats posed by human activities, this species is classified on the IUCN (The International Union for Conservation of Nature) Red List [3,4] as a vulnerable species. Bycatch is a major driver of population decline worldwide [5], and additional anthropogenic factors such as entanglement, boat strikes, infectious diseases, hooks, fishing lines, and plastic ingestion can also impact their lives [6,7]. Beyond human-related pressures, natural threats include predation and the impact of epibiont organisms; the latter can colonize the carapace and other body parts, hindering locomotion or impairing foraging efficiency [2]. These combined pressures often compromise the turtles’ ability to survive in the wild, making rescue and rehabilitation efforts essential components of conservation strategies. Consequently, many debilitated or injured individuals require extended hospitalization in specialized rescue centers, where they receive veterinary treatment, supportive care, and rigorous monitoring. During the rehabilitation period, potential stressors, such as periodic manipulation, life in tanks, limited movement, and artificial feeding, can influence the turtles’ well-being. Although these conditions are essential for clinical stabilization and pre-release preparation, they may represent additional challenges for individuals already compromised by prior injury or environmental stress. Rehabilitation aims to restore physiological balance, improve survival prospects, and ultimately contribute to conservation and management by maximizing the number of individuals successfully returned to their natural habitat [7,8]. Understanding how turtles physiologically respond to captivity is therefore crucial, as it allows clinicians to optimize husbandry practices and minimize stress during the recovery period.

During the rehabilitation period, the health and stress status of sea turtles can be monitored through the assessment of hematological, biochemical, and hormone parameters. Corticosterone (CORT) is the primary glucocorticoid hormone produced by reptiles in response to stressful stimuli [9,10], facilitating the maintenance of homeostasis. It is secreted from adrenocortical tissue, under the control of adrenocorticotropin released by the anterior pituitary: the hypothalamus releases corticotropin-releasing hormone, which stimulates the anterior pituitary to release adrenocorticotropic hormone. Elevated glucocorticoid levels promote gluconeogenesis, converting non-carbohydrate sources into glucose to meet increased energy demands [11]. Given its central role in the stress response, CORT is widely utilized as a physiological indicator of both acute and chronic stress in reptiles. In wildlife populations, stressors manifest in various forms, and the stress response often includes both physiological and behavioral modifications [12,13,14,15]. Furthermore, stress induces changes in the immune cells, which are essential for protecting the organism against infectious diseases and ectoparasites [16]. The evaluation of hematological parameters like differential leukocyte count on blood smears represents an alternative method for measuring stress due to rapid sampling and relatively cost-effective analysis [17,18]. Leukocyte profiles are particularly valuable in conservation physiology, as they are altered by stress and can be with circulating stress hormone levels [9]. Reptiles, like other vertebrates, have five categories of WBCs: lymphocytes, heterophils, eosinophils, basophils, and monocytes [12,19,20]. Notably, heterophil and lymphocyte counts are affected by stress in the opposite direction; researchers have often considered the ratio of one to the other as a composite measure of the stress response [17]. In addition, blood biochemical parameters play a key role in assessing the physiological condition of both individuals and populations, serving as diagnostic tools for the health status of sea turtles [12,21]. These markers are extensively used in rescue centers as they provide rapid, objective information regarding organ function, hydration status, nutritional condition, and metabolic balance.

Since previous studies have primarily evaluated individual biomarkers at isolated time points [2,8,10], the present work intended to explore whether the rehabilitation period facilitates a progressive reduction in physiological stress markers, thereby enabling individuals to re-establish homeostasis despite handling and confinement. Therefore, the aim of this study was to evaluate stress through hematological, biochemical and endocrine markers of sea turtles hospitalized at Centro di Referenza Nazionale sul Benessere, Monitoraggio e Diagnostica delle Malattie delle Tartarughe Marine (C.Re.Ta.M.) at the Istituto Zooprofilattico Sperimentale della Sicilia “A. Mirri” (Sicily, Italy). This study provides a novel longitudinal assessment of multiple physiological stress biomarkers in rehabilitating *C. caretta*, comparing juveniles and subadults across the early, mid-, and late phases of hospitalization.

## 2. Materials and Methods

### 2.1. Animals

Twenty-five loggerhead sea turtles (*C. caretta*) hospitalized at C.Re.Ta.M. were enrolled in this study. All specimens were housed in rehabilitation tanks where water quality parameters were rigorously maintained within the optimal physiological range for *C. caretta*. Furthermore, tank dimensions complied with the standards established by ISPRA [21] for sea turtle rehabilitation facilities. These controlled environmental conditions ensured consistent husbandry protocols throughout the study period, thereby minimizing external sources of physiological variability.

All the sea turtles were admitted to the Center for various clinical reasons, including fin injuries, hooks and fishing lines in their gastrointestinal tracts, or plastic ingestion. Carapace length (CCL) and weight were measured; based on these metrics, the animals were categorized into two age classes according to established criteria [21].

Twelve subjects were assigned to the juvenile group (21–40 cm CCL), and thirteen subjects were classified as the subadult group (41–65 cm CCL).

### 2.2. Blood Sample Collection

Blood samples were collected at three different time points: upon admission to the Center (T0), one month (T1) and two months (T2) post-admission. To account for circadian rhythmicity, samples were consistently collected in the morning, between 09:00 and 10:00, from the dorsal cervical sinus, using a 21-gauge needle attached to a 10 mL syringe, before being transferred into lithium heparin blood tubes.

Immediately after blood collection, smears were prepared and stained with Diff Quick, in accordance with the manufacturer’s instructions. According to Casal & Oros, 2007 [20], two-hundred leukocytes were counted and classified as lymphocytes, monocytes, eosinophils, heterophils, or basophils, and the heterophil–lymphocyte ratio (H/L) was calculated by dividing the total number of heterophils by the total number of lymphocytes observed within the 200-cell differential count. The H/L ratio was used as reliable indicator of physiological stress in reptiles. Blood samples were processed within 10–15 min of collection and centrifuged at 1200 rpm for 10 min to recover the plasma. Plasma for biochemical analyses was processed immediately after centrifugation. Plasma aliquots for CORT determination were stored at –20 °C until analysis.

Plasma CORT concentrations were determined using a corticosterone ELISA kit (Assay Genie CORTI UNEB0027, Colm and Sean, Dublin, Ireland). According to Miguel et al., 2020 [5], samples were diluted 1:40 in the provided assay buffer. Standard curves were generated as reported by the manufacturer. All samples were analyzed in duplicate, with absorbance measured at 450 nm using a microplate reader. The CORT concentrations were determined via interpolation from the standard curve and expressed in ng/mL.

Plasma concentrations of glucose (Glu), creatinine kinase (CK) and uric acid (UA) were measured using the multiparametric chemistry analyzer BS-480 Mindray (Li Xiting and Cheng Minghe, Shekou, Nanshan District, Shenzhen), an optical system (340 nm–800 nm).

Because in Italy capturing marine turtles exclusively for research purposes is prohibited by national and international regulations (EU Habitats Directive 92/43/EEC; CITES Appendix I), a wild-caught positive control group was not included. Nevertheless, the final sampling point (T2), performed shortly prior to release, served as an internal reference for physiological recovery.

### 2.3. Statistical Analysis

Descriptive statistics (mean, standard deviation, median and range) were performed using the Microsoft Excel (Microsoft Corporation, Redmond, WA 98052, USA).

Differences between categorical variables were assessed using the chi-square (χ^2^) test of independence, with the significance level set at α = 0.05. The normality of data distribution was assessed using the Shapiro–Wilk test. Since the raw data deviated significantly from normality, log-transformation was applied. Two-way repeated-measure analysis of variance (ANOVA) was applied on each parameter investigated to assess the differences due to the group and time points. Bonferroni’s test was applied for post hoc comparison.

Furthermore, Pearson’s correlation was used to determine whether corticosterone values were significantly correlated with hematological and biochemical results.

Data were statistically analyzed by the GraphPad Prism 5 software, and results were considered statistically significant at a *p*-value < 0.05.

## 3. Results

**Figure 1:**
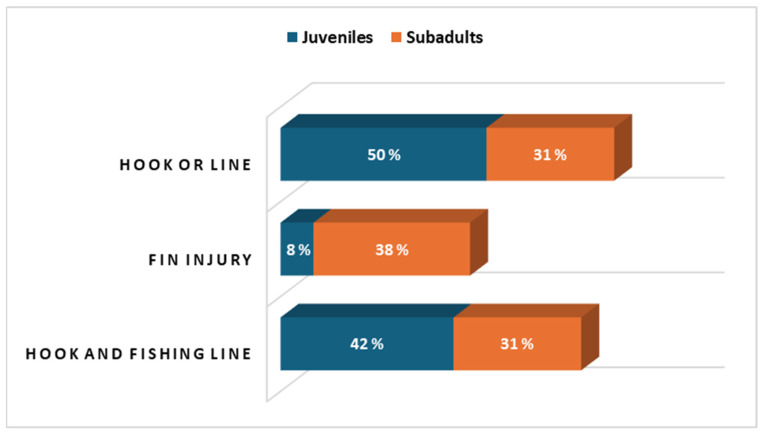
Percentage of hook and line ingestion, fin injuries caused by entanglement, and specific location of foreign bodies in juvenile (n = 12) and subadult (n = 13) subjects.

The 12 subjects included in the juvenile group exhibited a mean CCL of 30.6 ± 5.7 cm and a body weight of 4 ± 1.8 kg; the 13 subjects included in the subadult group presented a mean CCL of 52.5 ± 10.4 cm and a body weight of 17 ± 8.3 kg. All the sea turtles were recovering at the Center for different reasons; in particular, the primary cause of admission for the juvenile group was the ingestion of hooks or line (50%), whereas the majority of subadults had a fin injury by entanglement (38%). Thirteen (52%) of the twenty-five subjects had also ingested plastic debris. For these reasons, the hospitalized subjects had been recovering for a long time. The percentage distribution of hook and line ingestion, injuries to fins due to entanglement, and the presence of hooks in the esophagus or lines in the gastrointestinal tract for both juvenile and subadult groups is shown in Figure 1. The chi-square analysis revealed no significant differences in the distribution of injury causes between juveniles and subadults (χ^2^ = 3.15; df = 2; *p* > 0.05). Table 1 and Table 2 show the results obtained by applying descriptive statistics to the H/L ratio, CORT, GLU, CK, and UA at the three time points for each group, respectively.

Visual inspection of the raw data via boxplots revealed considerable dispersion across several hematological and biochemical parameters, particularly at T0 in both age classes. This variability was not attributable to statistical outliers; rather, it reflected genuine inter-individual differences associated with the heterogeneous clinical conditions of the turtles upon admission. All values were retained, as they were biologically plausible for rehabilitating sea turtles. Boxplots illustrating the distribution of each parameter across time points and both age classes are provided in Supplementary Figures S1 and S2.

**Figure 2:**
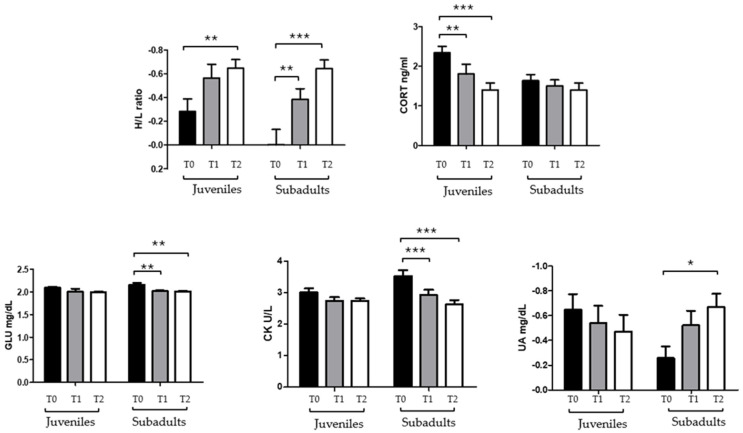
H/L ratio, CORT, GLU, CK and UA for different age classes and time points. The asterisk indicates the statistical significance used in GraphPad Prism 5 notation (* *p* < 0.05; ** *p* < 0.01, *** *p* < 0.001).

**Figure 3:**
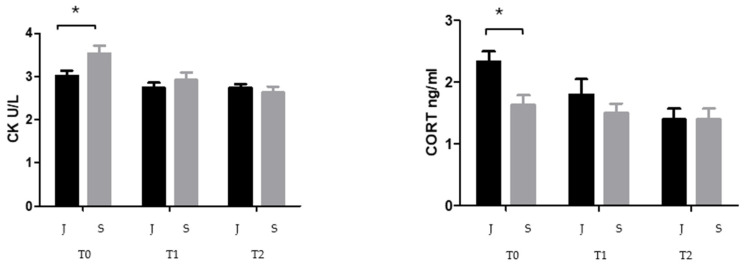
Comparison of CK and CORT plasma levels in the two different groups (J = Juveniles; S = Subadults). The asterisk indicates the statistical significance used in GraphPad Prism 5 notation (* *p* < 0.05).

Data were not normally distributed (Shapiro–Wilk test *p* < 0.05). Statistical analysis showed a significant effect of time on the H/L ratio (*p* < 0.0001), CORT (*p* < 0.0001), GLU (*p* = 0.0002), CK (*p* < 0.0001), and UA (*p* < 0.05), with a significative group x time interaction observed for CK (*p* = 0.016), CORT (*p* = 0.006) and UA (*p* = 0.035); however, no overall group effect was detected across the datasets. In the juvenile group, a significant decrease in the H/L (*p* < 0.01) was observed between T0 and T2, along with a reduction in CORT at both T1 (*p* < 0.01) and T2 (*p* < 0.001) compared to T0. In the subadult group, a significant decrease in the H/L ratio (*p* < 0.001), GLU (*p* < 0.01), CK (*p* < 0.001), and UA (*p* < 0.05) was recorded at both T1 and T2 compared to T0 (Figure 2). Two-way ANOVA further revealed significant age-related differences at T0. Specifically, CORT levels were significantly higher in juveniles than in subadults (*p* < 0.05), while CK concentrations were significantly elevated in subadults compared to juveniles (*p* < 0.05) (Figure 3). Furthermore, CORT showed no significant correlation with the hematological or biochemical parameters tested (*p* = 0.56) in either group.

## 4. Discussion

In this study the stress in *C. caretta* subjects hospitalized at C.Re.Ta.M was evaluated during the rehabilitation period by analyzing three time points: admission (T0), one month (T1) and two months post-admission (T2). Since stress physiology in reptiles is multifactorial, endocrine, hematological, and biochemical indicators were related within an integrated framework, considering their different temporal scales and physiological meanings.

One of the main physiological responses to stressful stimuli in vertebrates is the elevation of plasma levels of glucocorticoid hormones, such as CORT [9]. The results of this study show that juveniles had higher CORT concentration than subadults both upon admission and across sampling points.

This ontogenetic pattern is consistent with previous findings in which wild-caught juvenile loggerheads exhibited higher CORT levels than older age classes [13]. This is likely attributable to the heightened ecological stressors experienced during early life stages, including migration, environmental variability, and increased predation pressure [22].

In our dataset, most subjects of the juvenile group were admitted due to ingestion of hooks and fishing lines (Figure 1). Such injuries, known to cause substantial physiological and clinical stress [23], likely contributed to the elevated CORT observed at T0. The wide variability recorded at T0 reflects the diverse clinical presentations of the turtles upon arrival. Such dispersion is anticipated in wildlife rehabilitation contexts and represents the natural baseline variability inherent in compromised individuals.

During rehabilitation, the juvenile group showed a significant decrease in CORT levels at T1 and T2, eventually reaching values comparable to those of subadults. This trend indicates a reduction in acute stress once clinical stabilization and environmental predictability were restored. In contrast, the subadult group showed no significant variation in CORT concentrations over time. Moreover, the marked decline in CORT observed in juveniles during rehabilitation contrasts with the relative stability, suggesting that controlled clinical conditions may accelerate physiological recovery in juvenile individuals. This divergence highlights the importance of considering the rehabilitation environment as a distinct physiological context, rather than extrapolating directly from data on wild populations. The observed stability in subadults may reflect their specific life stage and greater physiological resilience: larger individuals typically possess greater energy reserves, more stable homeostasis, and reduced HPA axis reactivity [24,25].

CORT variations can be influenced by environmental, seasonal, nutritional and reproductive factors [10] as well as transportation [26]. Furthermore, levels can increase within minutes, indicating acute activation of the HPA axis [27,28,29].

For these reasons, hematological parameters like differential leukocyte count are essential for assessing health status over time.

Stress-induced leukocyte redistribution in reptiles typically involves an increase in heterophils and a decrease in lymphocytes [9,17]. This is due to leukocyte changes that occur more slowly than glucocorticoid fluctuations [23] and integrate stress exposure over days to weeks [9,30]. The heterophil–lymphocyte (H/L) ratio is considered a proxy of longer-term stress [17,18]. In this study, both age classes showed a decrease in H/L ratio during rehabilitation, consistent with improved health and reduced sustained stress [10,19]. Although our sample size (n = 25) may limit the detection of subtle associations, the absence of correlation between CORT and H/L ratio is physiologically expected and consistent with previous studies [10,18]. Although total white blood cell count can provide additional information, it is highly variable and influenced by factors unrelated to stress (e.g., hydration status or infections). In contrast, the heterophil-to-lymphocyte (H/L) ratio derived from differential leukocyte counts is considered a more stable and sensitive indicator of chronic stress in reptiles [9,30].

Consequently, their lack of correlation likely reflects intrinsic biological differences and natural variability within the sample rather than methodological limitations. Combining both markers provide a more comprehensive assessment of stress in *C. caretta* [31,32].

The decrease in H/L ratio observed in subadults is consistent with previous reports [10,19]; however, the results of this study deepen these findings by supporting the idea that hematological recovery occurs even in individuals with severe traumatic injuries, such as fin entanglement. This suggests that the H/L ratio is a sensitive indicator not only of generalized stress but also of clinical improvement during rehabilitation. The lack of correlation between CORT and H/L ratio in our dataset, although previously documented [10,18], is particularly informative here because it reinforces the idea that these markers capture different temporal dimensions of stress. These results, therefore, support the complementary use of hormonal and hematological indicators for clinical monitoring.

Blood biochemical parameters can vary across life stages due to differences in growth rates, diet, and metabolism [1], and are essential for evaluating an individual’s readiness for release [33]. This study focused on glucose (Glu), creatine kinase (CK), and uric acid (UA), which represent key biochemical stress markers in *C. caretta*.

Glucose (Glu) is a fundamental indicator of energy metabolism and can reflect nutritional status, environmental conditions, and stress [1,34]. In the subadult class, Glu levels decreased significantly at T2 compared to T0, consistent with the resolution of acute stress and stabilization of metabolic processes [10,35,36]. Although some authors reported a positive correlation between CORT and Glu [24,37], in this study this relationship was not observed. This is not unexpected: in clinical settings, glucose levels are strongly influenced by diet, temperature, and injury, which may obscure endocrine–metabolic correlations typically observed under controlled conditions.

Creatine kinase (CK) provides information on muscle damage [38]. Many individuals belonging to the subadult group (39%) presented fin injuries due to entanglement or boat collisions, with some requiring amputation. CK levels decreased significantly during rehabilitation, consistent with reduced muscle trauma, improved mobility, and adequate supportive care [10,38,39]. Consequently, CK levels in this context reflects tissue-level stress rather than endocrine activation. Although turtles gradually increase their voluntary activity during rehabilitation, their locomotor behavior remains constrained by the limited size of rehabilitation tanks, which do not allow continuous or high-intensity swimming comparable to natural conditions. Importantly, CK elevation in sea turtles is primarily associated with muscle damage, acute exertion, or traumatic injury rather than routine or moderate locomotor activity [40]. Many subadult turtles in this study presented traumatic injuries (e.g., entanglement or amputations), which likely contributed to the high CK values at admission. The progressive decrease in CK during rehabilitation therefore reflects tissue healing and reduced muscle trauma, rather than reduced activity [40].

Uric acid (UA) levels are influenced by renal function, hydration, fasting, and muscle catabolism. UA is also known to vary in response to physiological and metabolic stress, including oxidative stress and clinical compromise in reptiles [37,39]. In subadults, UA levels decreased during rehabilitation. However, because hydration status, renal function, and dietary intake were not directly measured, UA variations cannot be attributed to specific physiological mechanisms. Instead, the progressive decrease in UA is interpreted as a non-specific indicator of overall clinical improvement, consistent with patterns reported in clinically compromised sea turtles [39].

These comparisons indicate that biochemical recovery trajectories are influenced not only by injury type but also by age, clinical management, and environmental stability.

The absence of strong correlations among these markers is therefore physiologically coherent: they respond to different stimuli, operate on different timescales, and are modulated by distinct internal and external factors. This integrated approach provides a more complete understanding of the health trajectory of rehabilitating sea turtles and highlights the importance of evaluating multiple complementary indicators rather than relying on a single biomarker.

The sampling intervals adopted in this study were selected to monitor medium-term physiological recovery rather than short-term endocrine fluctuations. Monthly sampling aligns with standard clinical practice in sea turtle rehabilitation, as hematological and biochemical parameters typically shift over periods of days to weeks; therefore, high-frequency sampling would not provide meaningful additional data. Furthermore, more frequent blood collection would have necessitated repeated handling and restraint, both of which are potent stressors, potentially compromising both animal welfare and the clinical objectives of the rehabilitation process. Thus, the selected time points represent an appropriate balance between ethical considerations and the requirement to assess the integrated physiological effects of rehabilitation on stress. Moreover, standardized reference intervals for hematological, biochemical, and endocrine stress markers in *C. caretta* are not yet available, and published values show high variability depending on geographic origin, season, body size and health status.

A limitation of this study is the absence of a healthy free-ranging control group, which would have strengthened the comparative interpretation of the findings. However, this aspect must be considered in view of important ethical and legal constraints. In Italy, the capture of marine turtles exclusively for research purposes is prohibited under national and international regulations, including the EU Habitats Directive (92/43/EEC) and the Convention on International Trade in Endangered Species of Wild Fauna and Flora (CITES, Appendix I). Beyond these restrictions, the interpretation of stress-related biomarkers in *C. caretta* is further complicated by the lack of standardized reference intervals, which are known to vary substantially across geographic areas, environmental conditions, and sampling contexts. In this framework, a longitudinal within-subject design was adopted as the most appropriate and ethically compliant approach, allowing each individual to serve as its own control over time. Notably, the final sampling point (T2), collected shortly before release, can be reasonably interpreted as reflecting a condition of physiological recovery and thus represents the closest achievable proxy to a “healthy” state under rehabilitation conditions. While this approach does not fully replace the value of an external control group, it provides a robust alternative that minimizes inter-individual variability and aligns with current conservation and animal welfare standards.

For these reasons, stress responses were evaluated through a longitudinal within-subject design, comparing everyone’s physiological parameters across the rehabilitation period (T0–T2) and between age classes.

## 5. Conclusions

In conclusion, our results enhance our knowledge of stress responses in *C. caretta* housed within rehabilitation centers and suggest that the rehabilitation period does not appear to impose additional physiological stress. Conversely, the progressive normalization of endocrine, hematological, and biochemical parameters over time indicates that rehabilitation may support the recovery of general health status. This trend highlights the capacity of sea turtles to gradually re-establish homeostatic balance when provided with stable environmental conditions, adequate nutrition, and controlled handling procedures. The observed improvements also reinforce the value of structured rehabilitation programs as an essential component of conservation strategies for this vulnerable species. The contemporary monitoring of hematological, biochemical, and hormonal parameters provides a useful index of the health status of each subject during the rehabilitation period. These markers enable clinicians to detect subtle physiological changes, evaluate the effectiveness of therapeutic interventions, and make informed decisions regarding the timing of release. Importantly, the integration of multiple biomarkers offers a more comprehensive understanding of stress dynamics than any single parameter alone, supporting a multidimensional approach to health assessment in marine turtles.

However, further studies are needed to clarify the factors that influence stress variability in rehabilitating *C. caretta* and to better define species-specific physiological ranges. Future research should include larger sample sizes and longitudinal monitoring to track individual trajectories throughout the entire rehabilitation process. Experimental or controlled studies could help disentangle the effects of specific stressors (e.g., injury type, handling frequency, or environmental conditions) on endocrine and hematological responses. In addition, comparative analyses across different rehabilitation centers and across age classes would allow the identification of context-dependent patterns and improve the generalizability of stress indicators in *C. caretta*. Expanding collaborative networks among rescue facilities may also facilitate the development of standardized protocols for stress evaluation, ultimately enhancing the quality of care and the success of conservation efforts.

## Figures and Tables

**Table 1:** Mean ± standard deviation (SD), median, and range of corticosterone (CORT), heterophil–lymphocyte ratio (H/L), glucose (GLU), creatinine kinase (CK) and uric acid (UA) plasma concentrations in juvenile subjects.

Juveniles									
	T0			T1			T2		
Parameter	Mean ± SD	Median	Range	Mean ± SD	Median	Range	Mean ± SD	Median	Range
H/L	0.8 ± 0.8	0.405	0.2–3	0.4 ± 0.3	0.29	0.04–1.3	0.2 ± 0.1	0.25	0.07–0.5
Corticosterone (ng/mL)	317.9 ± 152.4	374	5.9–455	187.2 ± 182.2	130.2	1.7–400	8.5 ± 11.3	15.3	3.8–36.8
Glucose (mg/dL)	125.4 ± 24.1	123.5	86–155	110.2 ± 32.9	111.5	21–150	99.5 ± 13	101	84–115
Creatine Kinase (U/L)	149.7 ± 1197	1187.5	190–3548	758.9 ± 659.1	624	95–2601	705.3 ± 456.8	549	287–1868
Uric Acid (mg/dL)	0.4 ± 0.5	0	0–1	0.5 ± 0.5	0.5	0–1	0.5 ± 0.5	0.6	0–1

**Table 2:** Mean ± standard deviation (SD), median and range of corticosterone (CORT), heterophil–lymphocyte ratio (H/L), glucose (GLU), creatinine kinase (CK) and uric acid (UA) plasma concentrations in subadult subjects.

Subadults									
	T0			T1			T2		
Parameter	Mean ± SD	Median	Range	Mean ± SD	Median	Range	Mean ± SD	Median	Range
H/L	1.7 ± 2.1	0.9	0.2–7.4	0.5 ± 0.3	0.4	0.1–0.9	0.3 ± 0.2	0.2	0.1–0.7
Corticosterone (ng/mL)	82.2 ± 101.5	34.2	2.02–370	68.6 ± 105.7	31.2	4.1–387.5	62.4 ± 101.2	34.4	2-375
Glucose (mg/dL)	150.5 ± 40.7	140	91–204	104.7 ± 13.9	102	70–126	104.7 ± 10.9	107	90–122
Creatine Kinase (U/L)	9086.8 ± 1380.9	2306	564–44,509	1920.2 ± 2742	1035	94–9480	749 ± 964.9	501	100–3701
Uric Acid (mg/dL)	1.4 ± 1.3	1	0–5	0.5 ± 0.5	0.3	0–1.6	0.3 ± 0.4	0	0–1

## Data Availability

Data is contained within this article.

## Supplementary Materials

The following supporting information can be downloaded at: https://www.mdpi.com/article/10.3390/ani16101554/s1, Figure S1: Graphical representation of the distribution of each physiological parameter (H/L ratio, corticosterone, glucose, creatine kinase, uric acid) at T0, T1, and T2. The central line represents the median, while the box indicates the interquartile range (IQR), reflecting the central 50% of values. Wider boxes especially for corticosterone and creatine kinase, indicate higher inter individual variability, whereas glucose and uric acid display narrower IQRs. Whiskers extend to 1.5 × IQR, and points beyond them represent biologically plausible outliers. Figure S2: Graphical representation of the distribution of each physiological parameter (H/L ratio, corticosterone, glucose, creatine kinase, uric acid) at T0, T1, and T2 in sub-adult turtles. The central line represents the median, while the box indicates the interquartile range (IQR), reflecting the central 50% of values. Wider IQRs, particularly for corticosterone and creatine kinase, indicate greater inter-individual variability, whereas glucose and uric acid show more stable distributions. Whiskers extend to 1.5 × IQR, and points beyond them represent biologically plausible outliers.

## Abbreviations

The following abbreviations are used in this manuscript:

CORT	Corticosterone
Glu	Glucose
CK	Creatine Kinase
UA	Uric Acid
H/L	Heterophil–Lymphocyte Ratio
