# Supplementary material for: Stress Assessment in Caretta caretta During the Rehabilitation Period
Source: Animals (Basel). 2026 May 20;16(10):1554. doi: 10.3390/ani16101554 (PMC13203431; doi:10.3390/ani16101554)
Supplement: Supplementary file 1 [file animals-16-01554-s001.zip › animals-4235105-supplementary.pdf]

animals

Supplementary

Stress Assessment in *Caretta caretta* During the Rehabilitation Period

Chiara Lomonaco [1,2], Giorgia Schiró [1,2], Paola Galluzzo [2,] *, Rosaria Disclafani [2,3,] *, Irene Vazzana [2], Salvatore Dara [2], Giuseppe Piccione [1], Vincenzo Monteverde [2,] † and Claudia Giannetto [1,] †

[1] Department of Veterinary Science, University of Messina, 98168 Messina, Italy; chiara.lomonaco1@studenti.unime.it (C.L.); giorgia.schiro@izssicilia.it (G.S.); giuseppe.piccione@unime.it (G.P.); claudia.giannetto1@unime.it (C.G.)
[2] National Reference Center on Welfare, Monitoring and Diagnostics of Sea Turtle Diseases, 90129 Palermo, Italy; irene.vazzana@izssicilia.it (I.V.); salvatore.dara@izssicilia.it (S.D.); vincenzo.monteverde@izssicilia.it (V.M.)
[3] Center for Sustainability and Ecological Transition, University of Palermo, 90133 Palermo, Italy
* Correspondence: paola.galluzzo@izssicilia.it (P.G.); rosaria.disclafani@izssicilia.it (R.D.)
† These authors contributed equally to this work.

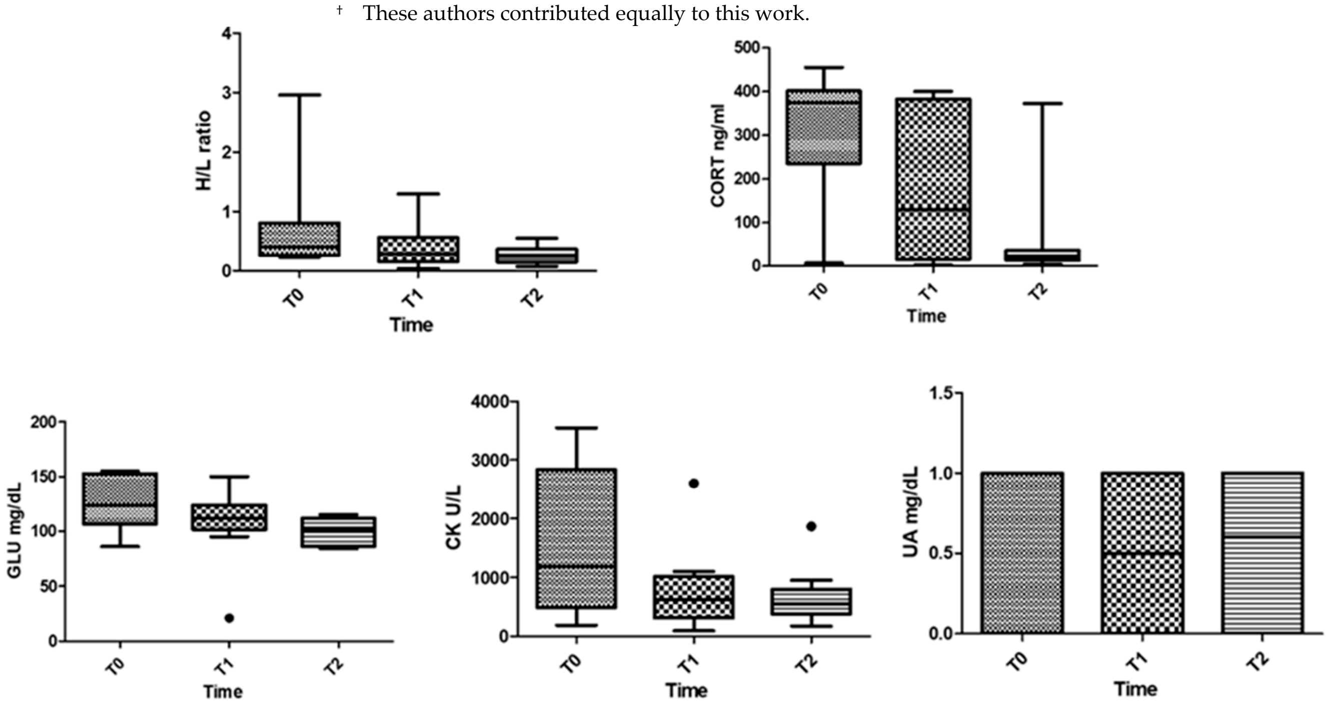

Figure S1: Graphical representation of the distribution of each physiological parameter (H/L ratio, corticosterone, glucose, creatine kinase, uric acid) at T0, T1, and T2. The central line represents the median, while the box indicates the interquartile range (IQR), reflecting the central 50% of values. Wider boxes especially for corticosterone and creatine kinase, indicate higher inter-individual variability, whereas glucose and uric acid display narrower IQRs. Whiskers extend to 1.5 × IQR, and points beyond them represent biologically plausible outliers.

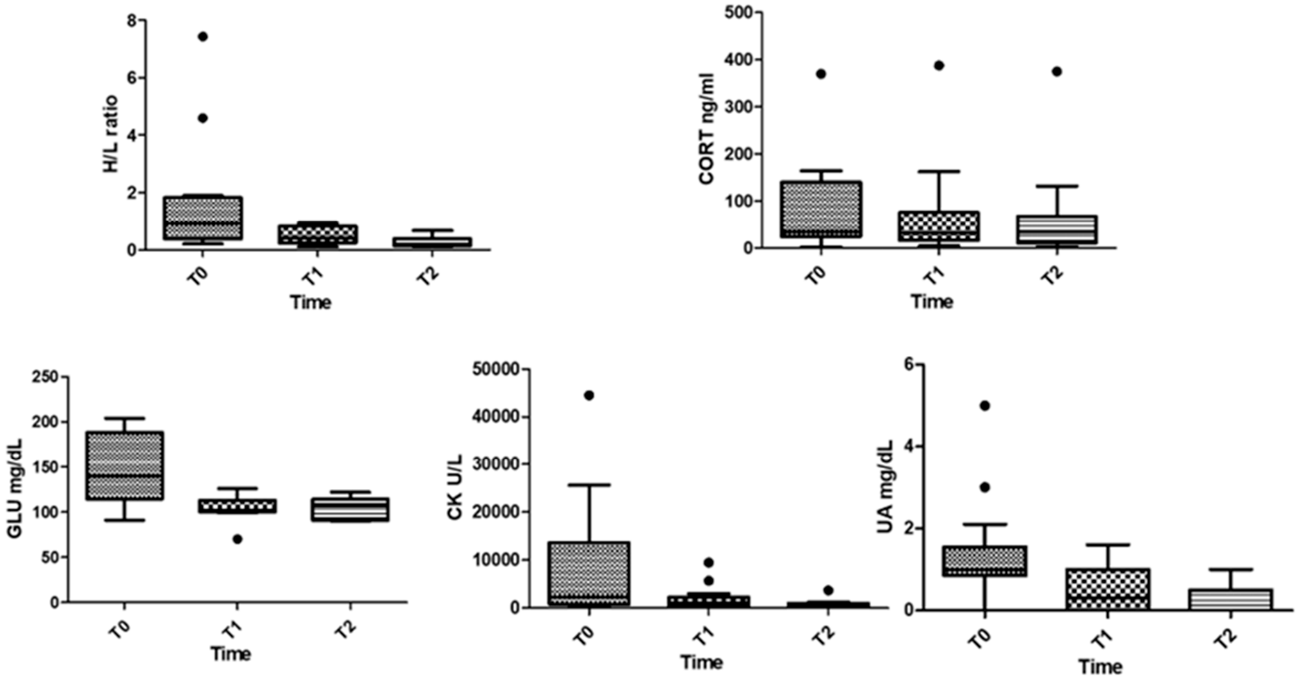

Figure S2: Graphical representation of the distribution of each physiological parameter (H/L ratio, corticosterone, glucose, creatine kinase, uric acid) at T0, T1, and T2 in sub-adult turtles. The central line represents the median, while the box indicates the interquartile range (IQR), reflecting the central 50% of values. Wider IQRs, particularly for corticosterone and creatine kinase, indicate greater inter-individual variability, whereas glucose and uric acid show more stable distributions. Whiskers extend to 1.5 × IQR, and points beyond them represent biologically plausible outliers.
